# DNA Methylation Patterns in the Round Goby Hypothalamus Support an On-The-Spot Decision Scenario for Territorial Behavior

**DOI:** 10.3390/genes10030219

**Published:** 2019-03-14

**Authors:** Vincent Somerville, Michaela Schwaiger, Philipp E. Hirsch, Jean-Claude Walser, Karen Bussmann, Alexandra Weyrich, Patricia Burkhardt-Holm, Irene Adrian-Kalchhauser

**Affiliations:** 1 Program Man-Society-Environment, Department of Environmental Sciences, University of Basel, Vesalgasse 1, CH-4051 Basel, Switzerland; vincent.somerville@unil.ch (V.S.); michaela.schwaiger@fmi.ch (M.S.); philipp.hirsch@unibas.ch (P.E.H.); karen.bussmann@unibas.ch (K.B.); patricia.holm@unibas.ch (P.B.-H.); 2 Genetic Diversity Centre Zurich, ETH Zurich, Universitätstrasse 16, CH-8092 Zurich, Switzerland; jean-claude.walser@env.ethz.ch; 3 Leibniz Institute for Zoo and Wildlife Research (IZW), Department of Evolutionary Genetics, Alfred-Kowalke-Str. 17, D-10315 Berlin, Germany; weyrich@izw-berlin.de

**Keywords:** *Neogobius melanostomus*, reproductive strategy, epigenetic mechanisms, brain, latent effects

## Abstract

The question as to how early life experiences are stored on a molecular level and affect traits later in life is highly topical in ecology, medicine, and epigenetics. In this study, we use a fish model to investigate whether DNA methylation mediates early life experiences and predetermines a territorial male reproductive phenotype. In fish, adult reproductive phenotypes frequently depend on previous life experiences and are often associated with distinct morphological traits. DNA methylation is an epigenetic mechanism which is both sensitive to environmental conditions and stably inherited across cell divisions. We therefore investigate early life predisposition in the round goby *Neogobius melanostomus* by growth back-calculations and then study DNA methylation by MBD-Seq in the brain region controlling vertebrate reproductive behavior, the hypothalamus. We find a link between the territorial reproductive phenotype and high growth rates in the first year of life. However, hypothalamic DNA methylation patterns reflect the current behavioral status independently of early life experiences. Together, our data suggest a non-predetermination scenario in the round goby, in which indeterminate males progress to a non-territorial status in the spawning season, and in which some males then assume a specialized territorial phenotype if current conditions are favorable.

## 1. Introduction

Life experiences influence the fitness of animals by shaping traits like survival, growth rate, or fecundity, and behavior [1]. For example, fish dispersal and migration behaviors [2,3] or their response to predators [4,5] depend on conditions during early life stages. Additionally, reproductive behavior is often shaped by experience. For example, the outcome of competitive or of mating interactions is not only shaped by immediate cues (such as the size of the opponent or the attractiveness of the mate), but also by early life experiences of food availability, predator pressure, social status or adverse experiences [6,7]. However, how such early life experiences are stored on a molecular level and later translated into gene expression patterns, morphological traits and behavioral phenotypes is not well understood.

The presence of alternative behavioral phenotypes within a single species, and the observation that early life experiences influence adult phenotypes, both suggest a role for epigenetic mechanisms. Epigenetic mechanisms are capable of storing early life experiences and translating them into a behavioral phenotype [8,9] because they can respond to environmental changes without affecting the DNA sequence, and can regulate gene expression [10,11].

The best studied epigenetic mechanism is DNA methylation. DNA methylation has been shown to respond to pregnancy in mice [12], temperature in wild vertebrates [13], numerous environmental factors in plants [14] and larval nutrition in bees [15]. DNA methylation at promoters [16], enhancer elements and in gene bodies [17] can regulate gene expression. Importantly, DNA methylation can be stable over mitotic cell cycles and is known to mediate cellular memory [18]. Indeed, DNA methylation patterns correlate with behavioral outcomes after early life stress in humans and rodents [19,20], or after toxicant exposure in zebrafish [21]. Thus, DNA methylation acquired earlier in life may, for example, silence a behavior-relevant gene and thus predispose the animal towards a certain behavioral phenotype later in life. Indeed, earlier studies in Atlantic salmon have highlighted the potential role of DNA methylation as the mediator between the environment and the reproductive phenotype [22]. However, our understanding of the role of DNA methylation patterns in predisposing individuals for the expression of certain phenotypes is limited. In particular, studies which assess whether DNA methylation patterns predict an animal phenotype prior to its manifestation are still missing.

Reproductive behaviors are usually associated with certain easily discernible phenotypes, which makes them an excellent model to study associated epigenetic mechanisms. Fish in particular display a great variety of reproductive phenotypes [23,24,25], such as external versus internal fertilization, paternal versus maternal brood care, one-to-one pairings versus broadcast spawning in aggregations, or hermaphroditism [24,26,27]. Fish reproductive phenotypes often entail characteristic colors and/or morphologies which are easy to detect. They are therefore among the best-studied experimental models within behavioral biology [28] and have been investigated in detail in many fish species [27,28,29]. Males in particular often adopt a territorial or a non-territorial phenotype. Typically, territorial or bourgeois males monopolize and defend resources such as nesting opportunities, court for females and provide brood care. Non-territorial males occasionally adopt a parasitic or sneaker life style [28,29] and try to steal fertilization by mimicking females and/or releasing sperm during the mating of a spawning couple [28,30]. Fish male reproductive phenotypes are most commonly conditional, i.e., dependent on life history events or ecological factors [31]. Prominent examples for alternative reproductive phenotypes are described within the families Gobiidae, Cichlidae, Centrarchidae, Salmonidae and Labridae [28,29]. In recent years, some of these male reproductive phenotypes have been associated with molecular phenotypes. Several studies report differential gene expression between brains of dominant and subordinate males, or reproductive and pre-spawning males, for example in salmon [32], peacock blenny [33], cichlids [34,35], ocellated wrasse [36,37], bluegill sunfish [38], black-faced blenny [39], and bluehead wrasse [40].

**Figure 1:**
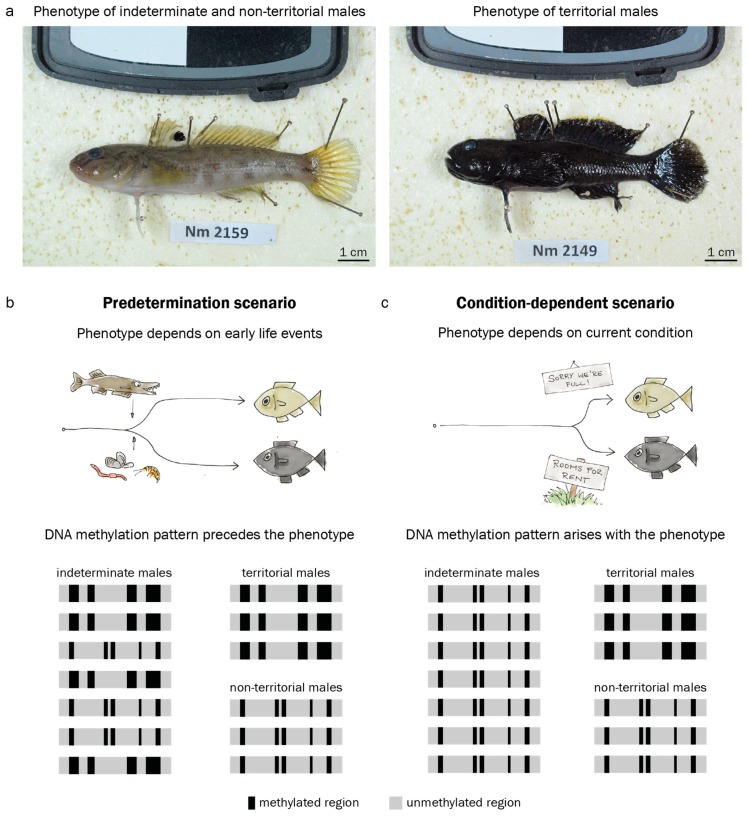
The male territorial phenotype in the round goby. (**a**). Usually, round goby males (and females) are light brown or grey. In the spawning season, territorial males develop a black coloration. (**b**). The territorial phenotype may be predetermined by early life conditions such as predation or food availability. If this predetermination is mediated by DNA methylation (DNAme), characteristic patterns should precede the appearance of the external phenotype. (**c**). Alternatively, the territorial phenotype may be induced by current conditions such as nest availability. In a condition-dependent scenario, characteristic DNAme patterns should arise concomitantly with the phenotype. Black and grey areas represent methylated and unmethylated regions in the genome.

The round goby *Neogobius. melanostomus* displays no major inter- or intra-sexual dimorphism outside the mating season. During the mating season, however, some males display a pale, inconspicuous coloring and do not guard nests, while others undergo morphological changes and develop an intense black body pigmentation and puffy cheeks (Figure 1a) [41,42]. The black-colored males occupy a nest, court females, and guard the eggs after oviposition [42]. Bleeker et al. (2017) [41] described morphological differences in size, gonado-somatic index and other morphological features between putative sneaker males and territorial males, with few intermediates present in the population. This may indicate that the round goby mating phenotype is determined before the first breeding season, and does not depend on current conditions. At the same time, Bleeker et al. (2017) [41] suggest that all males above a size threshold of 9.35 cm have the potential to eventually become territorial at some point during mating season, provided that nesting sites are available. Indeterminate males above the size threshold are most likely capable of, but not bound to become a nestholder. This may indicate that the round goby mating phenotype is induced by context (nest availability, male-male competition) and thus depends on current conditions. A conditional tactic for males of intermediate size is common in other goby species such as black goby, sand goby or grass goby [43,44,45]. Importantly, the study of epigenetic marks requires an assembled genome, which is available for the round goby [46]. The round goby is therefore an excellent model to study the role of epigenetics in the establishment of reproductive phenotypes.

To investigate a link between DNA methylation as a molecular mechanism regulating reproductive phenotype and the phenotype itself, investigations must focus on the functional organ controlling the phenotype. In the vertebrate brain, the hypothalamus regulates many aspects of male reproductive phenotypes [47]. It intersects the neuronal and the endocrinological networks, and controls gonad development and social aspects of reproduction through the hypothalamus-pituitary-gonad axis [48]. Inputs from sensory organs are received and integrated by the inferior hypothalamic lobes. Sexual maturation and the expression of reproductive phenotypes are then mediated by steroid hormones produced and released in the hypothalamus. For example, gonadotropin releasing hormone [49], arginine vasotocin as the major regulator of social reproductive behavior [50], KiSS peptides as triggers for gonadotropin-releasing hormone (GnRH) release [48], and brain aromatase [51] are produced in the hypothalamus.

**Figure 2:**
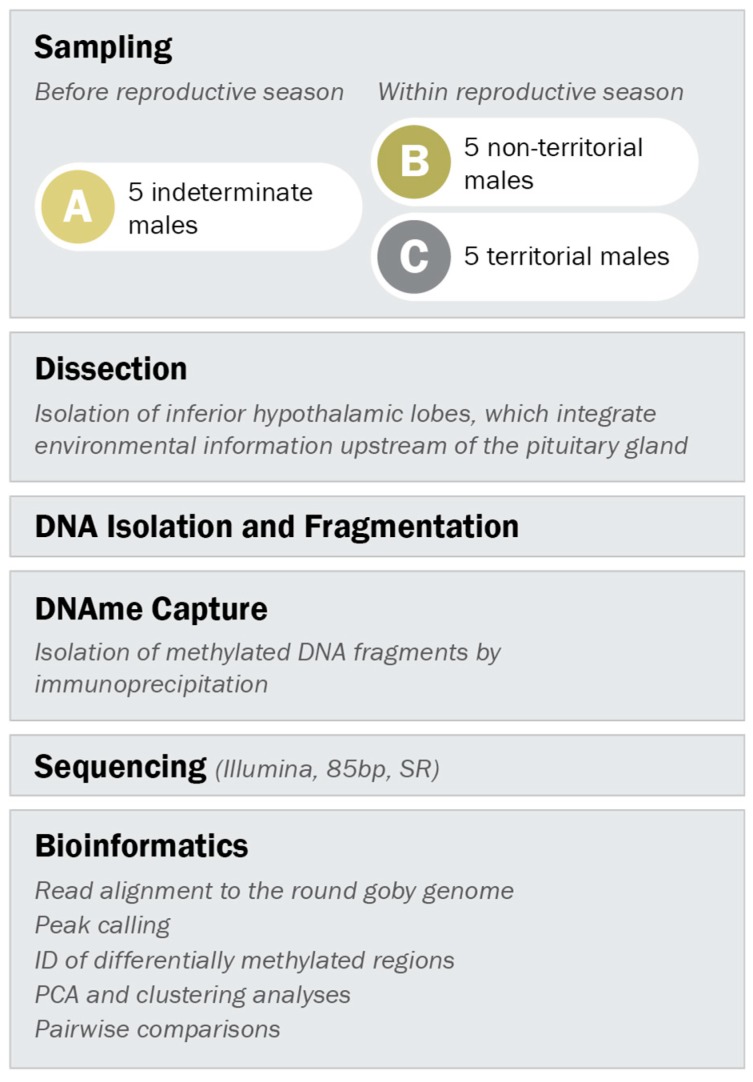
Experimental setup. Indeterminate males were sampled before the reproductive season, while males with non-territorial and territorial phenotype were sampled within the reproductive season. DNA methylation patterns in the inferior hypothalamic lobes were analyzed according to the outlined procedure.

In this paper, we investigate DNA methylation as a potential mechanism responding to early life experiences and regulating adult reproductive phenotypes in the round goby. We confirm that round goby reproductive phenotypes can be predisposed using growth back-calculations in territorial and non-territorial males. We map round goby brain regions by 3D reconstruction to confirm the location of the hypothalamus. Then, we analyze DNA methylation patterns in the inferior lobes of the hypothalamus and hypothesize (1) a predetermination scenario, where early life experiences would be epigenetically imprinted (Figure 1b), or (2) an on-the-spot decision scenario, where indeterminate males with baseline DNA methylation patterns would adopt a territorial phenotype only if conditions are favorable (Figure 1c). To this end, we sample phenotypically indeterminate males before the spawning season, and phenotypically territorial and non-territorial males during the spawning season. We identify territorial-like DNA methylation patterns and then investigate whether these patterns are present in the hypothalamus before the expression of the territorial phenotype. We assume that an appearance of territorial methylation patterns in indeterminate males caught before spawning season would suggest predetermination, while an appearance of territorial methylation patterns only upon overt phenotype expression would suggest a conditional determination (Figure 1b,c). In other words, if phenotypically indeterminate males exhibit territorial-like DNA methylation patterns, then predetermination of territorial behavior is more likely. If phenotypically indeterminate males do not exhibit territorial-like DNA methylation patterns, an on-the-spot scenario is more likely. Finally, to compare our DNA methylation data to available gene expression data from other fish species, we analyze promotor DNA methylation at genes whose orthologs are differentially expressed between male reproductive phenotypes in other fish species.

## 2. Materials and Methods

### 2.1. Growth in First Year and Luminosity

To investigate whether the territorial phenotype in the round goby could potentially be predisposed, we related growth rate in the first year of life to body pigmentation in adults using 113 males. Body pigmentation in fish is often controlled through endocrine pathways [52,53], and body pigmentation expressed as luminosity is a commonly used proxy for reproductive status in male round goby [41,42] (Figure 1a).

Males were sampled by hand netting and with fykenets in the Bay of Gdansk in Poland in the spring of 2009 (Table S1). Individuals were frozen after catch and later thawed prior to digital photography. To determine luminosity, each individual was photographed on its left side next to a grayscale for calibration. Relative luminosity was measured using Photoshop Elements 9 (Adobe systems, San Jose, CA, USA). To obtain an intuitive value for luminosity between 0 and 1 and to include both the white and black ends of the color spectrum into this value, the luminosity was expressed as 1 − (1/(White-Black) × (Fish-Black)). To determine growth in early life, operculum bones were analyzed. Each individual’s body length was measured as total length from the tip of the snout to the end of the caudal fin. The operculum bone was removed, boiled in water and cleaned for age and growth measurements. Age of each individual was measured by counting the winter bands on the opercula bone. The size of each individual in the first and second year of their lives was then calculated according to Bagenal and Teasch (1978) [54] using the equation: $\mathrm{Fish}\left(i\right)=\frac{Y*L\left(\mathrm{Oi}\right)*\left(L\left(F\right)-Y\right)}{L\left(\mathrm{OT}\right)}$, where L(Oi) is the length from the tip of the opercula to the i-th winter band, L(OT) is the total length of the opercula, L(F) is the length of the fish and Y is the y-intercept of the equation for the linear regression between L(OT) and L(F). Size at age one was then subtracted from the size at age two to acquire the growth in the first year. We used a Spearman’s R ranks test to detect a relationship between a trait value of early life (growth) and of adult life (pigmentation as a proxy for reproductive behavior).

### 2.2. Brain Morphology

To locate the hypothalamus, we reconstructed the morphology of the round goby brain. The brain of several adult males was exposed by dorsally opening the skull. The individuals were then immersed in 30 mL 4% formalin in 0.1 M phosphate buffer (Stock solution 0.5 M, pH 7.4: 54.5 g Na_2_HPO_4_ (anhydrous), 16 g NaH_2_PO_4_ (anhydrous), 1000 mL distilled water). The fixative was exchanged once on the following day. A few days later, the individuals were transferred to PBS for sample shipping. For reconstruction, brains were removed from the cranial cavity and stained with 0.5% cresyl violet containing 0.01% Triton X-100 for 5.5 h. They were then dehydrated in an ascending ethanol series (50%, 70%, 80%, 96%, each for 1 h). Embedding in methacrylate was done in three steps using the Technovit 7100 Kombipack (Kulzer GmbH, Wehrheim, Germany). After pre-infiltration for 3 h in a 1:1 mixture of 96% EtOH and Technovit 7100 and overnight incubation in the infiltration solution (100 mL Technovit 7100 + 1 g Hardener I), the brains were embedded with 15 mL infiltration solution + 1 mL Hardener II. One brain was then cut in caudal-frontal orientation into transverse sections at a thickness of 5 µm. The brain was then reconstructed by aligning stained brain structures between sections.

### 2.3. Sampling of Males for Methylated DNA Analysis

Three types of male designations are used throughout this study: indeterminate males, non-territorial males, and territorial males. (1) All males are externally indeterminate outside of the reproductive season, i.e., prior to late April and after late August in the study area. From September to April, they are brightly colored and morphologically very similar to females. (2) The term non-territorial males refers to males that retain a brightly colored, female-like phenotype after the onset of the reproductive season, i.e., after late April. These males are non-reproductive at the timepoint of catch, but they may become reproductive and territorial at a later timepoint, i.e., before late August. In principle, males above a size threshold of 9.35 cm have the potential to become territorial males [41], although they may be prevented from expressing this potential by lack of food, lack of nesting opportunities or lack of mate. (3) The term territorial males refers to reproductive males that display a black body color as well as distinct morphological alterations in their facial structures (puffy cheeks) and that monopolize a nest.

Non-territorial males actively forage for food and only occasionally seek shelter. Territorial males guard and defend a nest, which they do not leave until the eggs have hatched. Therefore, we use minnow traps to catch non-territorial males, and artificial nests (spawning traps) to catch black territorial spawning males.

Importantly, our own catch data collected across 6 years suggests that the vast majority of the male population >10 cm exhibits territorial behavior. This is based on the observation that these males disappear from the minnow trap catch during the spawning season, and re-appear in the catch after the spawning season (Figure S1). In other words, during the spawning season, most large males are not accessible to a catch method that requires free foraging – most likely, because they occupy and defend nests.

All three male types were caught in the harbour of Kleinhuenigen, Basel, Switzerland (47°35′14.8′′ N 7°35′36.2′′ E, see Figure S2 for map) and processed according to the workflow depicted in Figure 2. Indeterminate males were caught before the reproductive season (between 21st of March and 24th of March 2016) with minnow traps. Territorial and non-territorial males were caught within the reproductive season (between 17th of May and 6th of June 2016) with spawning traps (Figure S3) as described in Hirsch et al. 2016 [55] and with minnow traps, respectively. 

All fish were caught from a sampling depth of 3–4 m with permission GS 18-07-01 from the environmental department Basel-Stadt and permission 1022H from the animal welfare committee Basel-Stadt. Care was taken to pick similar-sized individuals with the aim to minimize confounding noise introduced by different ages. Average total lengths ± standard deviation were 10.54 ± 0.45 cm for indeterminate males, 10.22 ± 0.23 cm for non-territorial males, and 10.5 ± 0.29 cm for territorial males (not significantly different according to pairwise *t*-tests). This is a size range in which all males have the potential to perform territorial behavior [41]. We determined age by scale analyses according to Grul’a et al. 2012 [56], and found that all animals were between 2 and 3 years old. Animals were terminally anesthetized after catch with clove oil (conc. 40 mg/L in a 1:10 EtOH-dilution) according to best practice regulation from the local fishery authority, transported to the laboratory, and frozen at −80° until further processing.

### 2.4. Brain Dissection

To isolate the inferior hypothalamic lobes, males were thawed on ice. The brains were exposed by removing the dorsal head tissue and the skull bones, followed by severing of the optical nerves and the spinal cord. The brains were removed from the skull and placed ventral side up on a plastic container on wet ice. Under a stereo microscope, the hypothalamus was dissected, placed in a chilled FastPrep Lysing Matrix A tube (#116910050, MP Biomedicals, Santa Ana, CA, USA) containing 400 mg of beads, flash frozen in liquid nitrogen and stored at −80 °C. A step by step documentation of the dissection procedure is provided in the Supplementary Files (Figure S4).

### 2.5. DNA Isolation

To isolate DNA, frozen samples were lysed by bead beating in 500 µL lysis buffer (0.1 M Tris pH 8.0, 0.2 M NaCl2, 5 mM EDTA, 0.4% SDS) for 20 s at 4 m/s on dry ice using the Fast Prep-24™ 5G (MP Biomedicals). DNA was isolated from the lysate by standard phenol/chloroform extraction and ethanol precipitation. DNA concentration, quality, and integrity were assessed with a Fragment Analyzer (Agilent Technologies, Santa Clara, CA, USA). The presence of DNA methylation in the round goby was confirmed with methylation-sensitive restriction digests and bioinformatics (Figures S5 and S6).

### 2.6. Enrichment of Methylated DNA

To enrich for methylated DNA regions, we used the MethylMiner™ Methylated DNA enrichment kit (Invitrogen, Carlsbad, CA, USA) as previously described [57]. DNA samples were first sheared to a fragment size of 400 bp on a Covaris M220 Focused-ultrasonicator. Fragments smaller than 100 bp were removed using Agencourt AMPure© XP beads. Then, we immunoprecipitated methylated DNA fragments according to the manufacturer’s instructions.

### 2.7. Library Preparation and Sequencing

Sequencing libraries were prepared at the Genomics Facility Basel with the KAPA Hyper Prep Kit (Kapa Biosystems, Wilmington, MA, USA) following the manufacturer’s instructions. Residual adapters and adapter dimers were removed with Agencourt© AMPure© XP beads (Beckman Coulter, Brea, CA, USA). Finally, 15 barcoded DNA samples (from five indeterminate, five territorial and five non-territorial males) were pooled equimolarly for sequencing after quantification with the Quant-iT™ PicoGreen© dsDNA Assay Kit. The pooled sample was concentrated to 8 pM for NextSeq sequencing. Single-read sequencing was performed for 85 cycles with a NextSeq 500/550 v2 sequencing reagent kit (Illumina, San Diego, CA, USA). Base calling and demultiplexing was performed by the Illumina Casava (1.8.2) software. We obtained 15.673.477 ± 1.482.935 raw reads per sample (min 13.245.959, max 17.652.564). Reads are archived at SRA with BioProject ID PRJNA515617.

### 2.8. Read Cleaning and Alignment

To clean raw reads, we discarded all reads containing Illumina Trueseq adapter sequence using Cutadapt version 1.9.1 [58]. This approach was preferred over adapter clipping considering the relation of read length (86 bp) to Illumina Trueseq adapter sequences (50 bp). We then filtered for quality with prinseq version 0.20.4 [59] (minimum phred quality score ≥20, no non-nucleotide sequences, CG content ≤20% or ≥80%, no low complexity reads). In a next step we aligned the processed reads to the round goby reference genome V2 [46] end-to-end with bowtie2 version 2.2.9 [60]. Mapping quality filtering (cutoff 10) and SAM to BAM and BED file conversion was done with samtools version 1.2 [61] and bedtools version 2.25.0 [62]. Reads mapping in two locations with exact same quality were discarded. Of the raw reads (15.673.477 ± 1.482.935), 98.18 ± 0.56% were retained after adapter removal and quality filtering. 92.58 ± 0.62% of the trimmed and quality cleaned reads aligned to the genome.

### 2.9. Peak Calling

To determine methylated regions in the genome, we called peaks with MACS2 based on the reads of methylation pull-downs compared to reads of sheared but uncaptured input DNA in each sample individually. Peaks from all samples were then merged using bedtools, resulting in 334′511 peaks. The number of methylation reads in each peak and each sample was calculated using FeatureCounts, ignoring strand information and reads that had a quality score of less than 1. We then removed peaks on short and often repetitive scaffolds (less than 250 kb), thereby removing 9% of all peaks, and peaks on scaffold 364. Scaffold 364 collectively displayed extremely high differences between samples and was therefore excluded from further analyses. The most likely explanation involves structural sequence elements with above/below average DNA methylation levels that differ between individuals [63]. 1% of the peaks was removed because they contained zero reads in 2 or more samples, which was likely caused by indels in individual fish.

### 2.10. Princpal Component Analysis and Dendrograms

We used the prcomp function in R [64] to calculate a principal component analysis (PCA) on the normalized read counts (counts per million, cpm) across samples. We then filtered out 6458 peaks with very low read count by keeping only peaks that had a cpm >1 in more than 80% of samples in at least one group (*n* = 156,647 peaks). Finally, Voom normalization was performed to be able to identify differentially methylated peaks using Limma. To calculate dendrograms/heatmaps, we used the heatmap.2 function in R on the normalized counts of differentially methylated BC peaks (54 peaks with −log10 adjusted *p*-value > 0.1) or on the first 6 Principal Components of the PCA of all filtered peaks (on normalized counts). Since one of the five territorial males (sample C3) behaved as an outlier in both analyses (Figure S7), it was excluded, and analyses were re-run without this sample. The most likely explanation is that non-hypothalamic tissue was inadvertently included during the dissection of the C3 brain.

### 2.11. Pairwise Comparisons

To identify differentially methylated peaks, we used Limma for group comparisons, calculating fold changes and *p*-values, which were adjusted for multiple testing using Benjamini-Hochberg correction. Regions with below-average adjusted *p*-values were identified from the overall distribution of *p*-value versus fold change in R (Figure S8, Table S2). For comparison AB, we chose the cutoff 0.01, for comparison AC, 0.1, and for comparison BC, 0.015 to single out peaks with high fold change and lower-than-bulk *p*-value. Regions passing the cutoff in two contrasts were considered as being overlapping for the respective phenotype. Open reading frames overlapping with the peaks or in the vicinity were identified in the round goby genome browser and identified with NCBI protein blast against non-redundant protein sequences.

### 2.12. Analysis of DNA Methylation at Candidate Gene Promotors

To compare our results with previous studies, we searched the literature on fish alternative reproductive tactics and sex determination for genes that were reported as differentially expressed in alternative reproductive morphs by quantitative PCR, microarray analysis, or transcriptome sequencing [32,33,34,35,36,37,38,39,40,65,66]. Gene names were noted as reported in text, tables or figures and are listed in Table S3. To identify the corresponding genes in round goby, we first identified the zebrafish orthologue of a differentially regulated gene on ZFIN and retrieved the gene symbol. Zebrafish gene symbols were not retrieved if the gene name as reported in the literature had more than three hits on ZFIN (e.g., GABA receptor α), or if the reported gene did not yield any hits on ZFIN (e.g., pfkar2b). Using Ensembl BioMart, the zebrafish gene symbols were then matched with zebrafish stable gene IDs. When one gene symbol matched two stable gene IDs, one was arbitrarily retained. When a gene symbol could not be linked to a stable gene ID, which can happen because of a recent gene curation (for example, the gene *l1cama* was merged from two genes in 2016), the RefSeq ID of the gene was retrieved instead. Finally, zebrafish stable gene IDs were used to retrieve zebrafish protein sequences through Ensembl BioMart (unique records only), and zebrafish RefSeq IDs were used to retrieve zebrafish protein sequences through Batch Entrez (Table S3). Then, round goby orthologs of those zebrafish proteins were identified with Blast2GO [67].

The same approach was followed with candidate pathways and gene groups associated with alternative reproductive strategies in the literature, for example neuronal plasticity (Table S3). Pathways and functions reported in the literature were disregarded when they were very general (such as catabolic process) or when the process was much more closely related to processes other than reproduction and therefore would yield many unrelated genes (such as skeletal system development). Zebrafish genes associated with the respective keywords were identified on ZFIN, and processed as above to identify round goby orthologues.

To analyze DNA methylation at genes differentially expressed between fish reproductive phenotypes in other species, we determined the methylation levels (read counts determined using Feature Counts, cpm normalization as described above for peaks) at the promoters of these genes. Since promoters are not annotated in the round goby, we defined promoter regions as the proximal region 2 kb upstream the TSS [57,68] and verified the existence of methylated and unmethylated promoters by plotting read counts per 2 kb for gene bodies and for promoters (Figure S9). Using reciprocal Blast, we confirmed the round goby orthologues for the zebrafish protein sequences previously retrieved. Then we used the Limma package in R to perform a Romer analysis (a gene set enrichment analysis method based on rotation testing, which allows to perform this analysis with fewer replicates) on those orthologues to determine the competitive enrichment of DNA methylation at genes associated with male reproductive phenotypes.

## 3. Results

### 3.1. Adult Male Phenotype Is Related to Early Growth Rate

**Figure 3:**
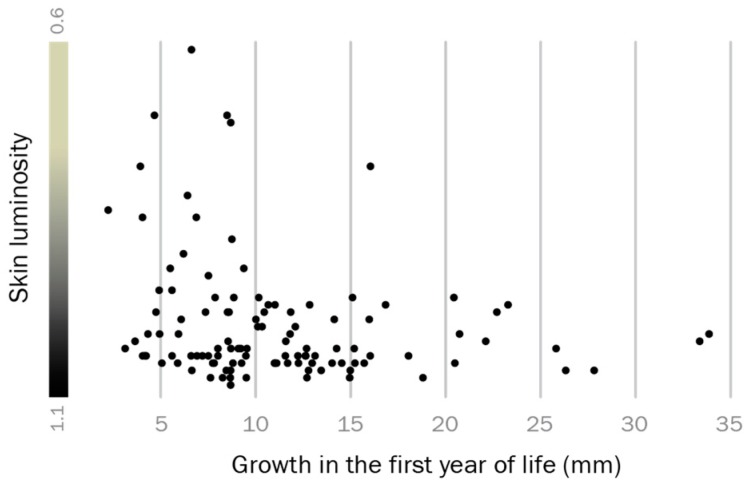
Predetermination of the territorial phenotype. Males that grew better than average in their first year of life (dots towards the right side of the graph) often display a black-colored territorial phenotype in the spawning season.

Growth rate analyses suggested that individuals which grow well in early life are more likely to express a territorial phenotype later in life. We expressed growth in the first year as back-calculated size increase in mm total length according to growth rings on opercula bones, compared it to whole body luminosity as a proxy for reproductive phenotype, and found that dark skin coloration is significantly associated with above-average growth in the first year of life (Spearman’s rank test: T = 0.018, Spearman’s R = 0.22, *p* = 0.018; Figure 3). Thus, adult reproductive phenotype and early life growth are related in the round goby.

### 3.2. The Round Goby Has a Typical Teleost Brain

**Figure 4:**
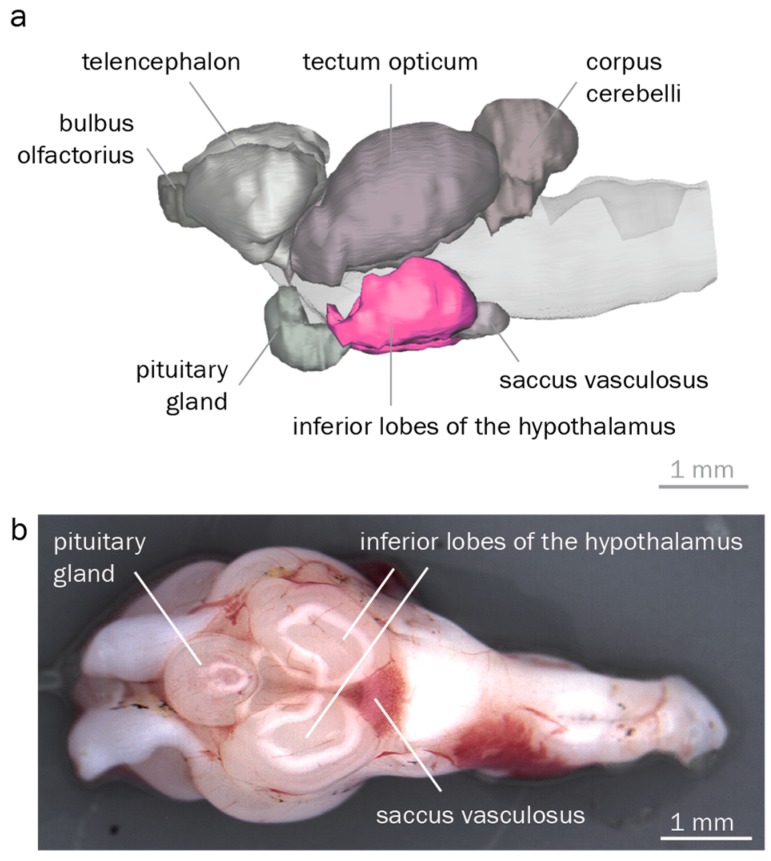
Round goby brain morphology. (**a**). 3D reconstruction of a round goby brain, lateral view. Inferior hypothalamic lobes are highlighted in pink. (**b**). Dissected round goby brain, ventral view. Anterior is to the left in both panels.

To reliably identify and dissect the hypothalamus, we reconstructed round goby brain morphology. Overall, we found a typical teleost brain characterized by a large rhombencephalon, a distinct visual tectum opticum, and pronounced inferior hypothalamic lobes. 3D reconstructions identified corpus cerebelli, tectum opticum, inferior hypothalamic lobes, telencephalon, saccus vasculosus, pituitary gland, and the bulbus olfactorius of the round goby (Figure 4). The cerebellum was the most conspicuous rhombencephalic structure. The tectum opticum covered most parts of the dorsal and lateral surface in the midbrain. The diencephalic components inferior lobes and pituitary gland dominated the ventral brain region. Measurements of the four major regions showed that the tectum opticum was the largest structure, followed by the telencephalon, the inferior lobes, and the cerebellum.

### 3.3. Territorial DNA Methylation Patterns Arise Concomitantly with the Phenotype

**Figure 5:**
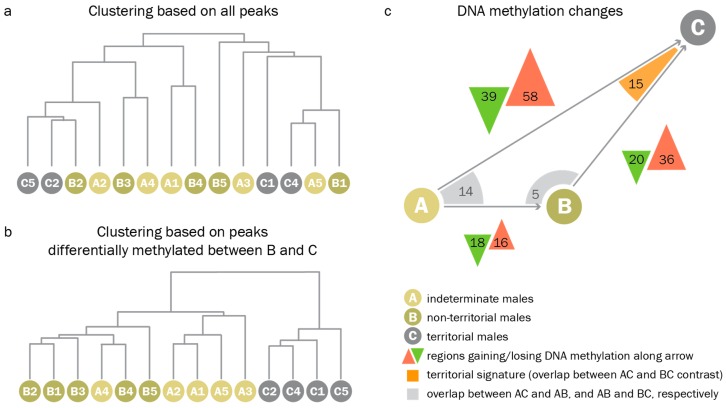
Indeterminate males do not display territorial DNA methylation patterns. (**a**). Clustering based on genome wide principal component analysis (PCA) eigenvalues based on read counts of all peak regions. Indeterminate, non-territorial, and territorial males are distributed randomly throughout the tree. (**b**). Clustering based on regions differentially methylated between non-territorial and territorial males. Branches for indeterminate, non-territorial, and territorial males cluster within the respective groups. Additionally, indeterminate males cluster with non-territorial males. (**c**). Results of pairwise comparisons. Green and red triangles represent regions that gain/lose DNA methylation during the transition represented by the grey arrow. Arrow lengths and triangle areas are drawn to scale to represent the number of regions with differential DNA methylation. Differentially methylated regions that overlap between two comparisons are indicated by orange/grey corners.

When clustering samples based on all peaks, we found that, globally, methylation patterns did not differ between reproductive phenotypes. Genome-wide hierarchical clustering of PCA eigenvalues derived from read counts at all peaks did not separate indeterminate, non-territorial, and territorial males (Figure 5a). We could, however, identify a set of differentially methylated regions (DMRs) between the different phenotypes in pairwise comparisons. When analyzing only those peaks which were differentially methylated between territorial and non-territorial males, we found that (1.) non-territorial males were most similar to indeterminate males, and (2.) territorial-like DNA methylation patterns were not present in any of the indeterminate males. When clustering all samples based on regions differentially methylated between territorial and non-territorial males, indeterminate males collectively clustered with non-territorial males (Figure 5b). The results of pairwise comparisons between indeterminate, non-territorial, and territorial males supported the idea of a stepwise progression from an indeterminate to a non-territorial, and, given permissive conditions, a territorial phenotype (Figure 5c). Indeterminate males and non-territorial males differed at only 34 regions, while non-territorial and territorial males differed at 56 regions, and indeterminate and territorial males differed at 97 regions.

Pairwise comparisons identified 15 regions with DNA methylation patterns that were characteristic for territorial males but not for indeterminate or non-territorial males (territorial signature). Using the round goby genome browser (access available on request) and the coordinates of those 15 regions, we found that these regions were frequently located in the vicinity of genes that were relevant for neuronal function and neural plasticity (Table S4). For example, genes associated with neuronal processes such as Tankyrase 1, sialic acid-binding Ig-like lectin, clarin 1, alkaline sphingomyelin phospho-diesterase, arachidonate 15-lipoxygenase, or neuroblast differentiation-associated protein AHNAK-like were overlapping or located close to some of the peaks. We also found genes potentially relevant for neuronal plasticity, such as genes implicated in signaling at membranes (star-related lipid transfer protein 8, an adhesion G-protein coupled receptor, and Guanine nucleotide exchange factor VAV2), genes regulating gene expression (THAP domain protein, several zinc finger proteins, WD repeat-containing protein 5, and bromodomain-containing protein 3), genes important for cell adhesion and extracellular matrix organisation (Tetraspanin, ADMTS-like protein, V-set and transmembrane domain containing protein), and genes encoding RNA regulatory proteins such as Staufen or the spliceosomal Gem-associated protein.

**Figure 6:**
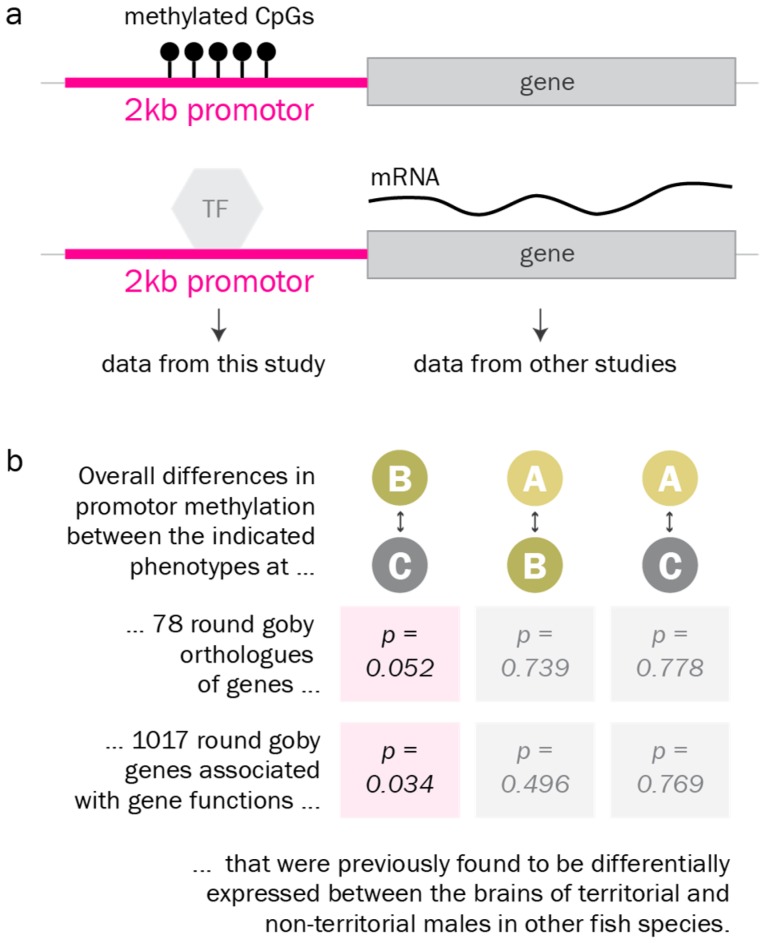
Promoter methylation of candidate genes. a. Promoter DNA methylation (balls on a stick) has been reported to regulate gene expression, for example by preventing transcription factors (TF) from binding to the promotor. b. According to Gene Set Enrichment analysis, promoters of candidate genes associated with reproductive phenotypes in fish in the literature (Table S3) are significantly more often differentially methylated between the territorial and the non-territorial phenotype than the average gene.

Finally, we investigated DNA methylation levels at the promoters of genes and gene groups that were previously reported as differentially expressed between alternative male reproductive phenotypes in other fish species [32,33,34,35,36,37,38,39,40,65,66]. We found that promoter methylation at these genes and gene groups differed from promoter methylation at the average gene. According to Gene Set Enrichment analysis, they were significantly more often differentially methylated between the territorial and the non-territorial phenotype than the average gene (Figure 6).

## 4. Discussion

In this study, we investigated whether brain DNA methylation may play a role in memorizing and translating early life experiences into a behavioral phenotype later in life in a wild fish. Specifically, we compared hypothalamic DNA methylation levels between male reproductive phenotypes in the round goby. We hypothesized that early life experiences could manifest epigenetically as DNA methylation patterns, which could then persist to adult life and predetermine reproductive phenotypes. Our alternative hypothesis was that DNA methylation patterns characteristic for a reproductive phenotype could be established at the same time as the externally visible phenotype (Figure 1b,c). We found that the reproductive phenotype in the round goby was linked to growth in the first year of life. We also found a clear distinction of territorial males based on hypothalamic DNA methylation pattern from non-territorial and indeterminate males. However, none of the indeterminate males resembled territorial males in their methylation patterns. Rather, hypothalamic DNA methylation reflected the respective current behavioral phenotype. This supports an on-the-spot decision scenario for the territorial phenotype, and also supports the idea that non-territorial males at the investigated size may represent an indeterminate group rather than being fixed on a non-territorial fate (Figure 1b, Scenario 2). Below we discuss the implications of these findings in relation to current research.

### 4.1. The Relationship between Early Growth and Adult Reproductive Phenotype

Research clearly shows that early life experiences can determine the phenotype at later developmental stages [69]. In line with these observations, our data on growth in the first year of life and later reproductive phenotype suggested a relationship between early life experiences and later reproductive phenotype in round goby. This supports the assumption that round goby reproductive phenotypes could potentially be predetermined by early life-conditions, and makes an early epigenetic setting plausible. In fish, growth rate is an essential fitness determinant [70]. Most fish show an indeterminate growth with limited capability of compensatory growth, and size at maturity can vary greatly [71]. Therefore, growth rate in the first year of life is a potentially powerful predictor of later reproductive strategy: once a male is outgrown by competitors, its ability decreases to monopolize a mating resource, which may promote the adoption of a non-territorial strategy. In addition to environmental determination, however, growth rate in fish may have a genetic basis and can also vary among populations. Research from fish populations in aquaculture [72], but also in the wild [73] suggest that growth rate differences can respond rapidly to altered selection gradients, and epigenetic variation can facilitate rapid adaptations to environmental changes. These results support the idea that a relationship between growth rate and other traits can evolve quickly in specific populations. In this specific case, growth/color data were obtained from a different population than methylation data. The fact that our growth back-calculations suggested predisposition, yet we did not find evidence for predetermination by epigenetic markers, may therefore be attributable to genetic variations in growth rate determination across different populations.

Another, equally likely explanation are differences in sampling strategies. The sample for the growth data was an ecological field sample that served as a first assessment of a relationship which we later analyzed by sampling very specific individuals. Thus, the field sample is an ecological population sample with individuals from a large size-spectrum, while the DNA-methylation study focused on a narrow size range. In the common goby *Pomatoschistus microps* or the black goby *Gobius niger*, only males at the ends of the size spectrum adopt a single phenotype, while males of medium size may act both as non-territorial and territorial males [42,74]. By focusing on a narrow and intermediate size range for DNA methylation analyses, we might have excluded the extreme ends of growth rates and, thus, predetermined individuals. However, restricting age and size in the molecular data set was essential to avoid confounding the results since age related changes in DNA methylation have been described [75].

### 4.2. Anatomical Identification of Brain Regions Controlling Reproduction

In vertebrates, the hypothalamus directs many aspects of male reproduction [47]. We therefore reconstructed round goby brain anatomy to unambiguously locate the hypothalamus. We find that the round goby displays a typical gobioid brain. Our brain map therefore enables us to reliably identify and isolate the inferior hypothalamic lobes. As is typical for gobies, the cerebellum and bulbus olfactorius are smaller, and the telencephalon larger than in other teleosts [76,77]. Some gobioid fishes, particularly those sieving substrates for food, rely heavily on internal gustation and therefore display an enlarged vagal lobe and an accompanying dorsal expansion of the rhombencephalon [77]. The round goby feeds primarily on benthic invertebrates [78] and lacks this feature. Finally, the round goby pituitary gland is larger than in zebrafish or rainbow trout [79,80] for reasons that are currently unknown.

Importantly, the round goby is a model organism for many aspects of behavior, such as auditory processing and communication through vocal calls [81,82], response to odors [83], sneaking [41,42], sex-biased movement and aggression [84,85], or feeding [86]. These behaviors are now amenable to neuromorphological and neurophysiological investigations given that the major brain regions have been mapped in this study.

### 4.3. Assessment of DNA Methylation Across Male Reproductive Phenotypes

While growth data suggest that the territorial reproductive phenotype may be linked to early life conditions, DNA methylation patterns in the hypothalamus suggest a condition-dependent scenario. Indeterminate and non-territorial males displayed similar baseline DNA methylation before and during the spawning season, and were clearly distinct from territorial males (Figure 5b). Territorial patterns were absent from all indeterminate males in the dataset. This suggests that territorial-like DNA methylation in the hypothalamus develops concomitantly with the appearance of the phenotype, and that territorial DNA methylation patterns in the hypothalamus are absent before the overt onset of the phenotype. Alternatively, predetermination could depend on a few key genes involved in regulating reproductive strategy (Figure 5c). Higher sample numbers would be needed to address this.

All in all, however, our results do not show that hypothalamic DNA methylation plays a role in the long-term memory of early environmental conditions in the round goby. Rather, our data supports an on-the-spot decision scenario (Figure 1c). This is interesting in the context of human mental health, where DNA methylation has been proposed to serve as a molecular memory mechanism altered by early life trauma and to correlate with mental health state. For example, childhood trauma and abuse induce characteristic DNA methylation patterns of the glucocorticoid receptor gene [87]. Also, DNA methylation levels at the Brain-Derived Neurotrophic factor gene are associated with Major Depressive Disorder [88].

Our data also indicate that the territorial phenotype may involve a tissue specialization process. In pairwise comparisons with territorial males, increases in methylation levels are more common than decreases in methylation levels (Figure 5c). Depending on the genomic position, increasing methylation can both silence [16] and activate gene expression [89,90] and is a hallmark of cellular differentiation and specialisation [91,92]. Therefore, the territorial phenotype may potentially represent a specialization achieved by channeling of gene expression.

Finally, it is important to note that the hypothalamus contains a high diversity of neurons [93,94]. Zones of proliferation and apoptosis have been described [95,96], also in the context of reproduction [97]. Teleost brain plasticity may therefore have an impact on global methylation patterns in the inferior lobes. What we have identified as territorial patterns may reflect an increase in a certain neuronal subpopulation rather than DNA methylation changes in existing cells.

### 4.4. Identification of Differentially Methylated Genes Involved in Reproductive Phenotypes

Previous studies identified distinct gene expression patterns associated with reproductive phenotypes in fish [32,33,34,35,36,37,38,39,40,65,66]. Similarly, we identified DNA methylation patterns that discriminate between non-territorial and territorial males. Some of the differentially methylated regions reside within predicted genes, others reside in the vicinity of predicted genes. It is an interesting observation that many genes physically close to differentially methylated regions seem to have neuronal functions.

However, statements on functions should be taken with a grain of salt in the absence of functional data for three reasons. (1) In novel genomes, gene function is inferred solely from orthology with human genes. This process is particularly unreliable in fish considering their potential for neofunctionalization after genome duplication [98]. (2) Also, DNA methylation (as most epigenetic marks) may affect genes at a distance [99]. Physical vicinity between a methylated region and a gene is therefore not necessarily functionally significant. (3) Finally, the functional role and significance of DNA methylation in fish has not been entirely clarified, and its effect on gene expression is not understood. Algorithms developed on mammals fail to identify CG islands in fish [100], and fish differ from mammals with respect to the distribution of methylated CGs in the genome [101]. Also, methylation patterns of exons and introns in zebrafish suggest that a gene’s transcriptional state strongly impacts gene body methylation in fish [102]. The observed differential methylation patterns may thus represent a consequence rather than a cause of transcriptional activity. This interpretation is in line with the observation that DNA methylation in the hypothalamus reflected current rather than past or future behavioral status.

These caveats notwithstanding, we find that genes previously found to associate with reproductive phenotypes in fish are, as a group, differentially methylated between territorial and non-territorial males in the round goby. In the future, concomitant investigations on brain DNA methylation and brain gene expression in the same individual could help clarify the impact of DNA methylation on gene expression in the context of male reproduction. Time course analyses may also help to clarify the dynamics of acquisition and erasure of epigenetic memories. We expect that a certain proportion of males in the analyzed size group has transitioned through a reproductive phase in the previous year. Yet, indeterminate and non-territorial males behaved homogenously as a group based on hypothalamic DNA methylation data. This suggests that they either share the same reproductive history, or that they have erased methylation patterns related to the previous territorial state in the hypothalamus. Epigenetic memories may, of course, persist in other parts of the brain, or in other tissues.

## 5. Conclusions

In conclusion, we find that the reproductive phenotype in the round goby is linked to growth in the first year of life. However, our data indicate that the territorial phenotype in round goby is not induced by pre-existing DNA methylation patterns in the inferior hypothalamic lobes. Rather, hypothalamic DNA methylation patterns align with the current phenotypic status. The methylation data suggest a stepwise progression from indeterminate male to non-territorial male to territorial male during spawning season. In this scenario, all indeterminate males progress to become non-territorial males in the spawning season. These, in turn, will specialize into territorial males if conditions are favorable.

## Supplementary Materials

The following are available online at https://www.mdpi.com/2073-4425/10/3/219/s1, Supplementary Files contain 8 supplemental figures. Figure S1: Most males become territorial. Figure S2: Map of sampling site; Figure S3: Sampling equipment; Figure S4: Inferior lobe dissection protocol; Figure S5: Experimental confirmation of DNA methylation; Figure S6: Bioinformatic confirmation of DNA methylation; Figure S7: Outlier identification. Figure S8: Volcano plots; Figure S9: Differential promoter methylation; Table S1: Growth and luminosity data from the Bay of Gdansk; Table S2: Peak data; Table S3: Candidate genes and candidate processes linked to alternative reproductive strategies in fish from the literature; Table S4: Blast results.
