# Supplementary material for: DNA Methylation Patterns in the Round Goby Hypothalamus Support an On-The-Spot Decision Scenario for Territorial Behavior
Source: Genes (Basel). 2019 Mar 14;10(3):219. doi: 10.3390/genes10030219 (PMC6471186; doi:10.3390/genes10030219)
Supplement: Supplementary file 1 [file genes-10-00219-s001.zip › genes-408701_supplementals revised_final.docx]

**Supplemental Files**

**DNA methylation patterns in the round goby hypothalamus support an on-the-spot decision scenario for territorial behaviour**

Vincent Somerville, Michaela Schwaiger, Philipp E. Hirsch, Jean-Claude Walser,

Karen Bussman, Alexandra Weyrich, Patricia Burkhardt-Holm, Irene Adrian-Kalchhauser

**Table of Contents:**

| **Figure S1. Most males become territorial.** | Page 1 |
| --- | --- |
| **Figure S2. Map of sampling site** | Page 2 |
| **Figure S3. Sampling equipment** | Page 3 |
| **Figure S4. Inferior lobe dissection protocol** | Page 4 |
| **Figure S5. Experimental confirmation of DNA methylation** | Page 5 |
| **Figure S6. Bioinformatic confirmation of DNA methylation** | Page 6 |
| **Figure S7. Outlier identification** | Page 7 |
| **Figure S8. Volcano plots** | Page 8 |
| **Figure S9. Differential promoter methylation** | Page 9 |


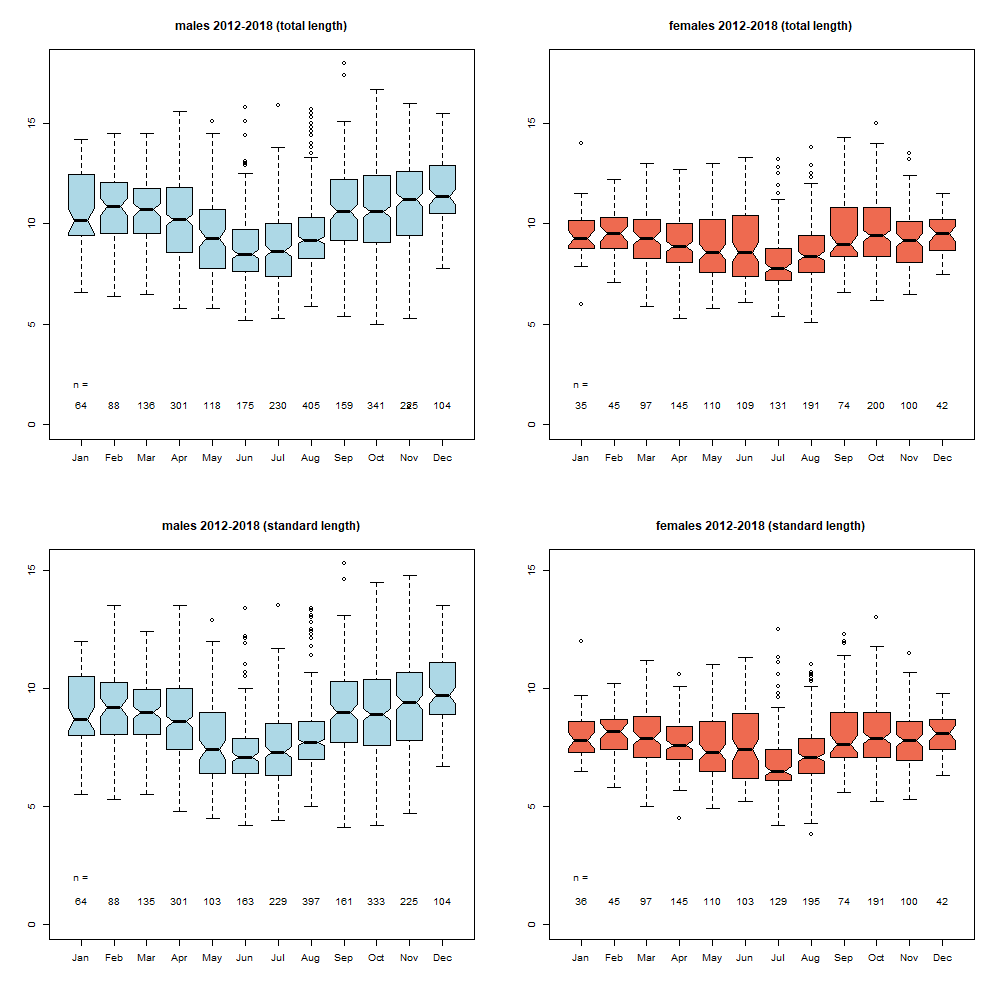


**Figure S1. Most large males become territorial in the reproductive season.**Boxplots of male total length by month across the years 2012 to 2018 derived from own unpublished minnow trap catch data. Larger males cannot be caught with minnow traps (which require free ranging behaviour) during the reproductive season, which lasts from late April to August in the study area. They reappear in the minnow trap catch after the reproductive season. This data structure indicates that a substantial proportion of large males occupies a nest and exhibits territorial behaviour during the season.


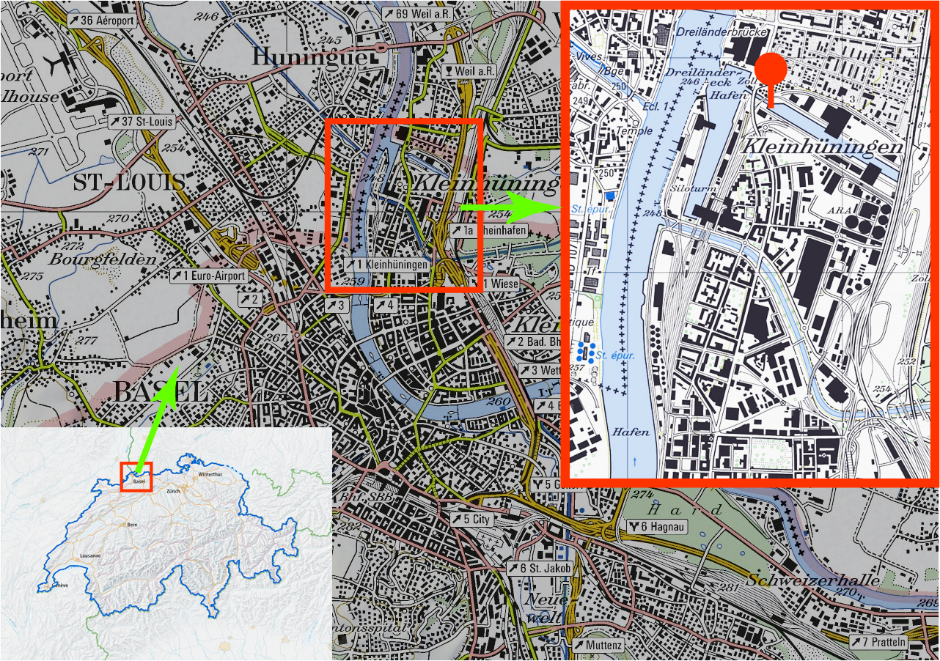


**Figure S2. Map of sampling location.**The sampling site (red pin) is situated in the river Rhine in Basel, Switzerland. The Rhine is the longest European river connecting Switzerland, Germany, Lichtenstein, France and the Netherlands and a key transport and trade route in Europe. The left small map represents the country Switzerland. The background map and the right map are closeup perspectives of the harbour Kleinhuningen in Basel and indicate the exact position where male gobies were trapped for investigation. Maps are downloaded from the public database SchweizMobil (<http://www.schweizmobil.ch>).

**Figure S3. Minnow traps and Spawning traps**.

Non-territorial and indeterminate round goby males were trapped using minnow traps (A) and B)) connected to a) a string for moving the trap. The trap contains b) a tea strainer to hold frolic dog food as bait and c) a rock to weight and stabilize the trap. Spawning traps (C) and D)) were used to sample territorial males. The trap consists of a bicycle basket (17 cm x 37 cm x 18 cm) fitted with d) hinged e) PVC tubes and covered with a net. c) A rock weights and stabilizes the trap.


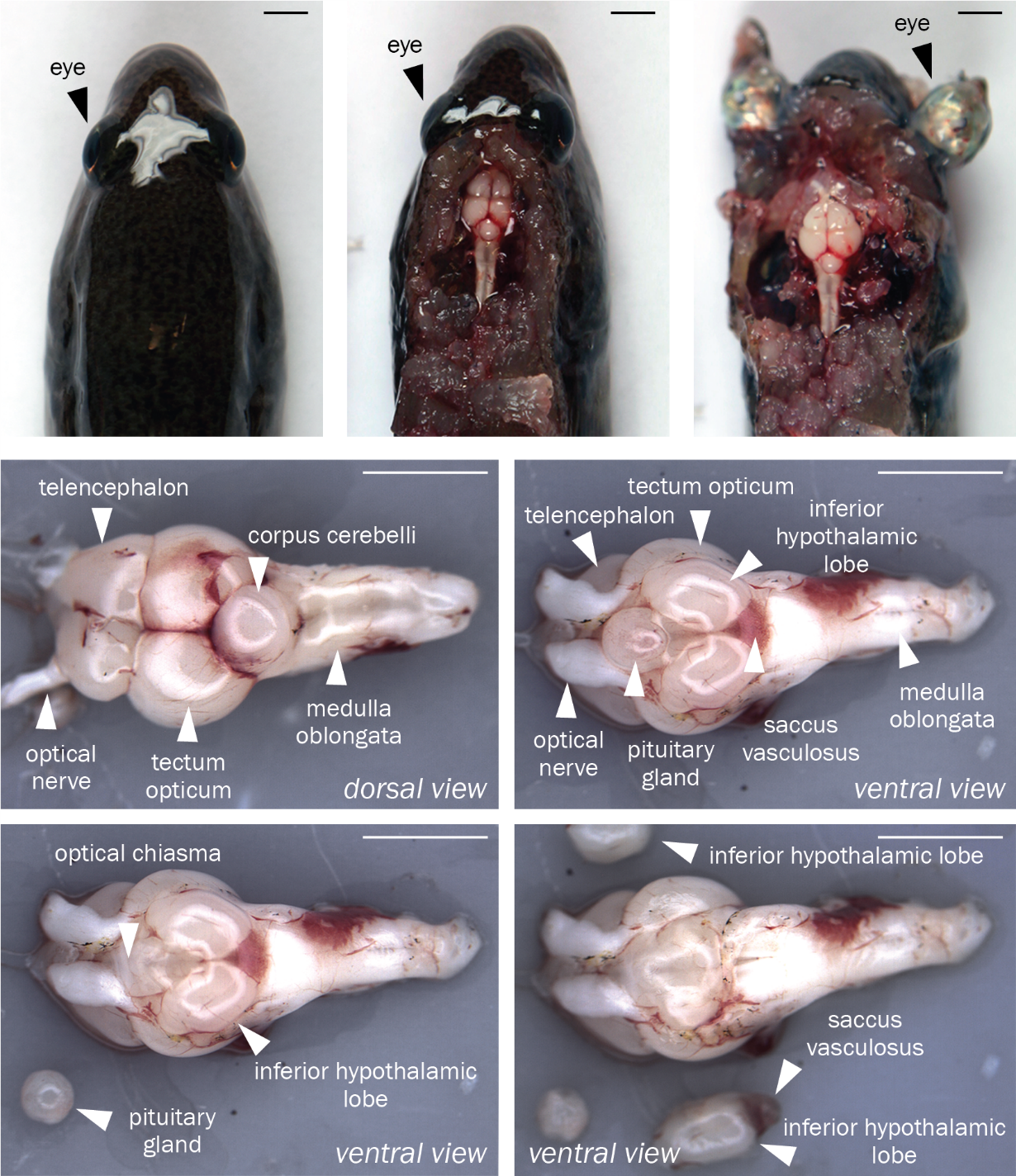


**Figure S4. Inferior hypothalamic lobes dissection protocol.** Top row: Dorsal view of round goby head – preparing the brain by removing skin cover and skull – exposing neuronal connection to the eyes for dissection. Middle row: Dorsal view of the whole brain – ventral view of the whole brain. Bottom row: Pituitary removed to access inferior lobes – inferior hypothalamic lobes removed. Scale bars: 2 mm.


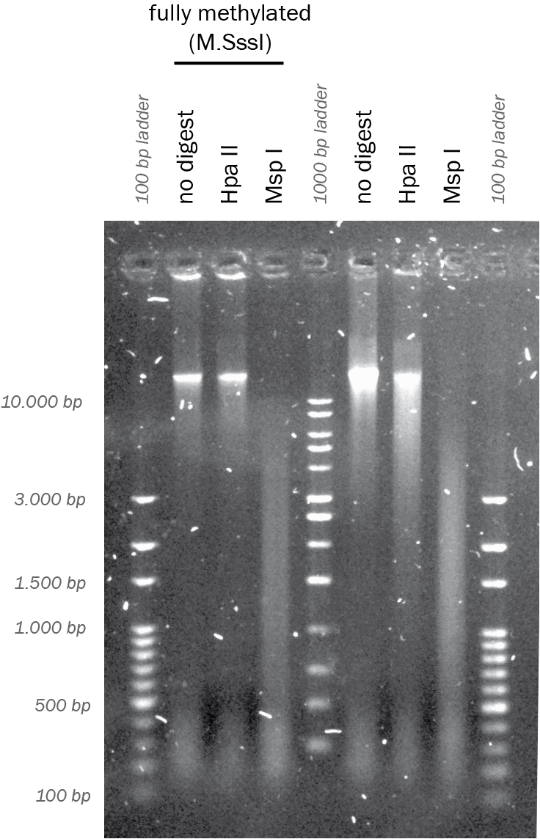


**Figure S5. Experimental confirmation of DNA methylation in the round goby.**

Fully methylated (left) and non-treated (right) genomic DNA was digested with two isoschizomeric restriction enzymes, the methylation sensitive enzyme Hpa II and the methylation non-sensitive enzyme Msp I. Both enzymes recognize the same sequence, but only Msp I can cut in the presence of DNA methylation. Hpa II digest was prevented by full methylation, but was also largely inhibited in non-treated DNA. The methylation-insensitive enzyme Msp I produced low molecular weight fragments irrespective of DNA methylation. DNA treated with the M.SssI methyltransferase is fully methylated and can be cut only by Msp I but not Hpa II, and therefore constitutes a useful control in this experiment.

| Dinucleotide | Occurrences in the genome | Observed frequency | Observed / Expected frequency |
| --- | --- | --- | --- |
| AA | 96338156 | 0.0959 | 1.5356 |
| AC | 59709807 | 0.0594 | 0.9517 |
| AG | 63361165 | 0.0631 | 1.01 |
| AT | 74056145 | 0.0737 | 1.1804 |
| CA | 74471577 | 0.0741 | 1.1871 |
| CC | 45743687 | 0.0455 | 0.7291 |
| CG | 25241469 | 0.0251 | 0.4023 |
| CT | 63336609 | 0.0631 | 1.0096 |
| GA | 57578712 | 0.0573 | 0.9178 |
| GC | 45751264 | 0.0455 | 0.7292 |
| GG | 45711105 | 0.0455 | 0.7286 |
| GT | 59574149 | 0.0593 | 0.9496 |
| TA | 65076644 | 0.0648 | 1.0373 |
| TC | 57588599 | 0.0573 | 0.9179 |
| TG | 74301466 | 0.074 | 1.1843 |
| TT | 95896645 | 0.0955 | 1.5286 |

**Figure S6. Bioinformatic confirmation of DNA methylation in the round goby.** CG dinucleotides are underrepresented in the round goby genome. This phenomena is observed in all organisms with DNA methylation because methylated cytosines are prone to deamination and thus mutation (CG 🡪 TG).


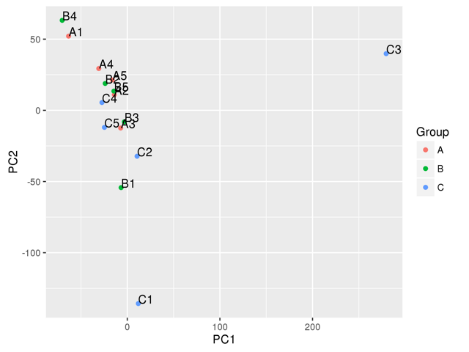


**Figure S7. Outlier identification.**

PCA based on the read couts of all filtered peaks. Sample C3 behaves as an outlier.

**
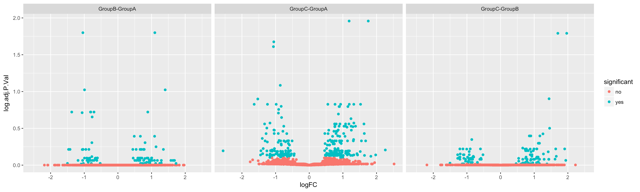
**
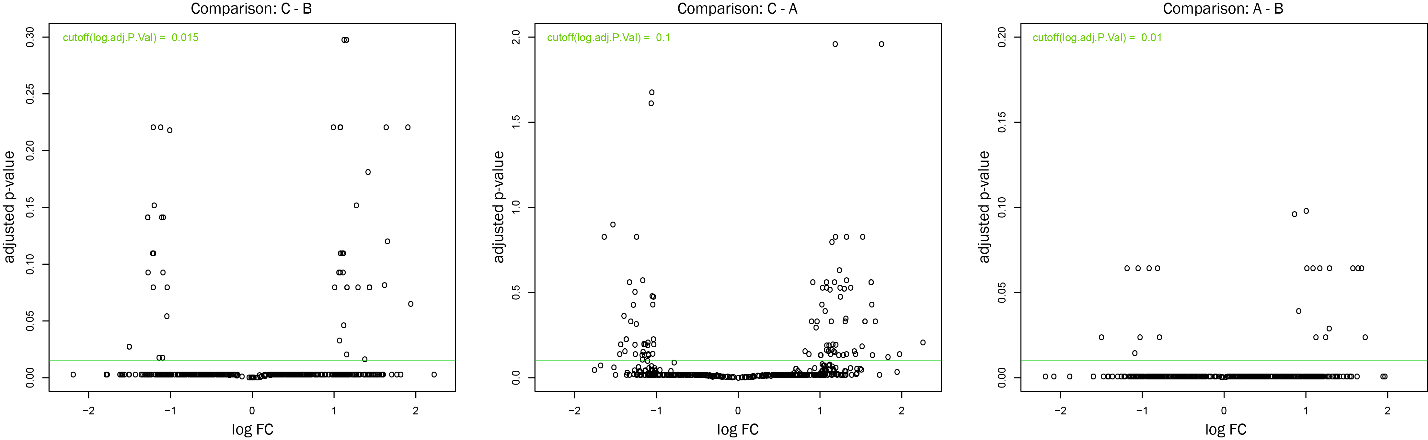


**Figure S8. Volcano plots.** Volcano plots are useful to identify regions with both high fold change and low adjusted (log) p-values. Cutoffs were chosen based on the overall distribution of the data at a similar overall location in the data cloud in the three pairwise comparisons (top row). Bottom row: Datapoints plotted to same-scale x-axes. The different distribution of the data cloud (which warranted customized cutoffs at similar data cloud locations rather than at a fixed log.adj.P.Val) becomes apparent. Points are colored according to above the cutoff (green) and below the cutoff (red) as defined in the top row.


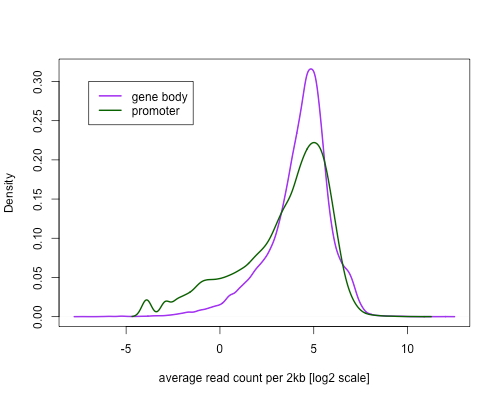


**Figure S9. Differential promoter methylation.** Read counts per 2 kb were calculated for gene bodies (magenta line) and promoters (2 kb upstream of putative TSS, green line). Gene bodies are, as expected for a fish, mostly methylated, as indicated by a single peak around 2^5 reads per 2 kb. Promoters can be methylated just like gene bodies are (right peak), but a substantial fraction of promoters is also unmethylated (peak shoulder at 0 reads per 2 kb), or in an intermediate methylation state.
